# Hybrid motion artifact detection and correction approach for functional near-infrared spectroscopy measurements

**DOI:** 10.1117/1.JBO.27.2.025003

**Published:** 2022-02-24

**Authors:** Lin Gao, Yuhui Wei, Yifei Wang, Gang Wang, Quan Zhang, Jianbao Zhang, Xiang Chen, Xiangguo Yan

**Affiliations:** a Xi’an Jiaotong University, State Key Laboratory of Manufacturing Systems Engineering, Xi’an, Shaanxi, China; b Xi’an Jiaotong University, School of Mechanical Engineering, Xi’an, Shaanxi, China; c Xi’an Jiaotong University, Key Laboratory of Biomedical Information Engineering of Education Ministry, School of Life Science and Technology, Xi’an, Shaanxi, China; d Xi’an Jiaotong University, National Engineering Research Center of Health Care and Medical Devices Xi’an Jiaotong University Branch, Xi’an, Shaanxi, China; e Massachusetts General Hospital, Harvard Medical School, Department of Psychiatry, Charlestown, Massachusetts, United States

**Keywords:** functional near-infrared spectroscopy, artifact detection, artifact correction, hybrid approach

## Abstract

**Significance:**

Functional near-infrared spectroscopy (fNIRS) is a promising optical neuroimaging technique, measuring the hemodynamic signals from the cortex. However, improving signal quality and reducing artifacts arising from oscillation and baseline shift (BS) are still challenging up to now for fNIRS applications.

**Aim:**

Considering the advantages and weaknesses of the different algorithms to reduce the artifact effect in fNIRS signals, we propose a hybrid artifact detection and correction approach.

**Approach:**

First, distinct artifact detection was realized through an fNIRS detection strategy. Then the artifacts were divided into three categories: BS, slight oscillation, and severe oscillation. A comprehensive correction was applied through three main steps: severe artifact correction by cubic spline interpolation, BS removal by spline interpolation, and slight oscillation reduction by dual-threshold wavelet-based method.

**Results:**

Using fNIRS data acquired during whole night sleep monitoring, we compared the performance of our approach with existing algorithms in signal-to-noise ratio (SNR) and Pearson’s correlation coefficient (R). We found that the proposed method showed improvements in performance in SNR and R with strong stability.

**Conclusions:**

These results suggest that the new hybrid artifact detection and correction method enhances the viability of fNIRS as a functional neuroimaging modality.

## 1 Introduction

Localized neural activity is accompanied by hemodynamic oscillations caused by the metabolic activity and concurrent electrical activity. The electrical firing requires more oxygenated blood supply to activate brain areas, causing an increase in local oxygenated hemoglobin and a decrease in deoxygenated hemoglobin. It results in an underlying link between electrical events and hemodynamic oscillations, which is generally known as neurovascular coupling.^1^^–^^3^ As an inexpensive and portable optical neuroimaging technique, functional near-infrared spectroscopy (fNIRS) can noninvasively monitor brain function by simultaneously measuring the concentration of changes of cerebral oxyhemoglobin (${\Delta [\mathrm{HbO}}_{2}]$) and deoxyhemoglobin (Δ[Hb]).^4^^–^^7^ It has attracted increasing attention during recent years. A major advantage of fNIRS over neuroimaging techniques, such as functional magnetic resonance imaging and positron emission tomography, is that fNIRS is portable. Therefore, fNIRS technique is easily applied to long-term measurements, which are also much more likely to exhibit frequent movement and produce motion artifacts in comparison with the short-term experiments. For example, an increased prevalence of movement for long-term monitoring can induce artifacts in fNIRS data. Rapid head shaking introduces great amplitude and high-frequency fluctuation in fNIRS signals, whereas slow head rotation will cause slow sustained varying oscillation along with lasting baseline shift (BS).^8^ Failure to correct for the artifacts may lead to biased or spurious conclusions.^7^ Therefore, the motion artifacts are still problematic for fNIRS monitoring, particularly in long-term recordings with additional challenges to be addressed in comparison with the short-term experiments. And it is necessary to apply preprocessing approaches to eliminate the artifacts for fNIRS signal collection.

Some artifact correction approaches have been proposed for fNIRS measurements. However, each suffers from some drawbacks. For example, the simplest way is to discard the data segments polluted by the artifacts.^9^ This approach is only suitable if the number of motion artifacts detected is low and the number of trials is high. However, it may not be ideal in cases where there are only a small number of trials. Adaptive filtering can be used for motion correction if the external signals are highly sensitive to motion artifacts but not to brain activity, such as an accelerometer. The drawback is that this approach cannot completely remove motion artifacts if the motion information in the external signals is not exact.^10^^–^^12^ Robertson et al. compared different methods including adaptive filtering, wavelet filtering, ICA,^13^ and linear regression, for removing motion artifacts from the measured signal, and claimed that the best motion artifact removal results were produced by ICA and regression. However, all these methods made use of the co-located channels to identify the signal that was related to motion.^14^ Furthermore, the wavelet-based (WB) methods could deal with motion spikes but exacerbate BS artifacts.^15^^,^^16^ Correlation-based signal improvement can effectively remove large spikes caused by head motion and improve signal quality and spatial specificity. The method assumes that oxy-Hb and deoxy-Hb are perfectly negatively correlated and become positively correlated only when there is an artifact, although this assumption might not always be true during certain developmental stages or under abnormal brain physiology.^7^ Metz et al.^17^ presented an acceleration-based artifact reduction algorithm with data adaptive threshold specifically designed for long-term measurements. Accelerometer-based motion artifact removal (ABAMAR) can be easily adapted to various monitoring fNIRS applications for correcting baseline motion artifacts, with a drawback of the application of global threshold for the standard deviation (SD) of the amplitude or concentration signals.^8^ Frank et al.^6^ reported a temporal derivative distribution repair procedure to discard BS and spike artifacts. However, this technique mainly corrects the low-frequency signal, and it is still necessary to develop an approach to deal with the high-frequency component in fNIRS signals.^6^ A spline interpolation method was proposed to identify and correct the BSs and smoothing methods to correct the spikes.^5^ However, it might be difficult to define a global threshold of the maximum amplitude change for finding BSs, and many parameters need to be adjusted manually, such as signal-to-noise ratio (SNR) and the degree of spline function. Scholkmann et al. developed a method based on moving SD and spline interpolation to reduce movement artifacts. However, a limitation is that the variances of the motion artifacts have to be greater than that of the physiological signal to ensure the correct reduction of the artifacts.^18^ Yucel et al. applied an approach in EEG applications to the applications of specialized NIRS optical fibers using improved optode-scalp coupling through miniaturized optical fiber tips. A potential limitation is that the position of the collodion-fixed fibers might introduce a bias.^19^ Brigadoi et al. compared the performance of different motion correction techniques on real data during a cognitive task, and revealed wavelet filtering is the most effective approach.^20^ Principle component analysis, spline interpolation, wavelet analysis, and Kalman filtering approaches were also compared using the accuracy of the recovered and simulated hemodynamic response function. The results showed spline interpolation produced the greatest improvement in mean-squared error, and wavelet analysis generated the greatest increase in contrast-to-noise ratio.^21^ Thus each algorithm has its own advantage. However, how to combine the advantage of these approaches is still an issue. Regarding this, Hu et al.^22^ demonstrated that the combined use of wavelet and moving average was preferable to each method on its own, whereas the combination of wavelet with targeted principal component analysis did not outperform wavelet alone.^23^ However, the moving average technique cannot be considered a motion correction method such as wavelet since it served a high-pass filter function to remove slow drifts. Di Lorenzo et al. proved that the combination of spline and wavelet outperformed its individual use using infant semi-simulated data. However, motion artifacts in infant data greatly differ from adults’ both in amplitude and frequency of occurrence.^24^ The artifact correction recommendations derived from infant and children data might not be generally optimal for all kinds of fNIRS data. Thus every approach mentioned above has its own advantages and weaknesses.

WB methods can provide good localization property resulting in isolation of abrupt changes for the motion artifacts from hemodynamic changes. They are very effective in removing high-frequency spikes as well as low-frequency and low-amplitude artifacts but not in removing BSs.^16^ On the other hand, spline interpolation can properly correct BSs by modeling the motions and subtracting from the original signal; however, it cannot deal with high-frequency spikes.^5^ An optimum artifact removal method should be capable of detecting the artifacts objectively and correcting different types the artifacts efficiently. Here, we proposed a hybrid approach in this study for artifact detection and correction for fNIRS measurements, gathering the strengths of the existing approaches. The method first detects the artifacts through fNIRS detection strategy. The artifacts are divided into three categories: BS, slight artifacts, and severe artifacts. Finally, the artifacts are corrected based on the categories by three main steps: severe artifact correction by cubic spline interpolation, BS removal by spline interpolation, and slight artifacts reduction by dual-threshold WB method. In order to compare the proposed approach with other algorithms, we detected and corrected the artifacts in the fNIRS data acquired during whole night sleep monitoring using different methods. The metrics, SNR and Pearson’s correlation coefficient (R), were applied for comparison.

## 2 Materials and Methods

### 2.1 Hemodynamic Signals Extraction

In this study, the hemodynamic signals were calculated from the optical signals acquired by fNIRS multiparameter collectors. $\Delta [{\mathrm{HbO}}_{2}]$ and Δ[Hb] were estimated using the modified Lambert–Beer Law^25^ and the equations can be expressed as

$$\Delta [{\mathrm{HbO}}_{2}]=\frac{\alpha_{1}\Delta {\mathrm{OD}}^{\lambda_{1}}+\alpha_{2}\Delta {\mathrm{OD}}^{\lambda_{2}}}{L},$$ (1)

$$\Delta [\mathrm{Hb}]=\frac{\beta_{1}\Delta {\mathrm{OD}}^{\lambda_{1}}+\beta_{2}\Delta {\mathrm{OD}}^{\lambda_{2}}}{L},$$ (2)

where L represents the measuring depth of the fNIRS probes, and coefficients $\alpha_{1}$, $\alpha_{2}$, $\beta_{1}$, and $\beta_{2}$ in the equations vary with the wavelength of near-infrared light. The wavelengths of our fNIRS measurements are 735 and 850 nm, and the corresponding values of the coefficients are $\alpha_{1}=330.1717$, $\alpha_{2}=-131.3958$, $\beta_{1}=-127.6967$, and $\beta_{2}=184.5598$, respectively.^26^

### 2.2 Hybrid Motion Artifact Detection and Correction

**Fig. 1:**
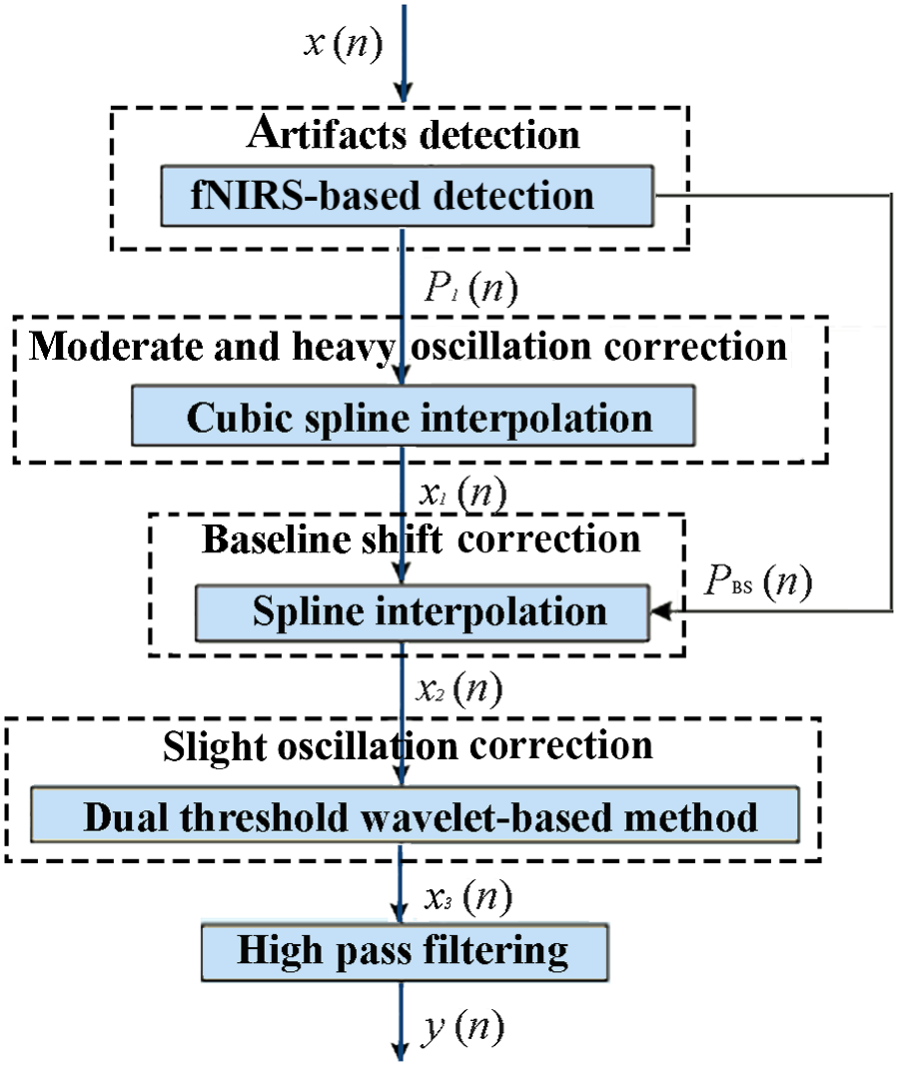
Flowchart of the proposed method. x(n): original fNIRS signal containing the artifacts. $x_{1}(n)$: fNIRS signals processed by severe oscillation correction. $x_{2}(n)$: $x_{1}(n)$ processed by BS correction. $P_{I}(n)$: information of different oscillations. $P_{\mathrm{BS}}(n)$: information of BS. $x_{3}(n)$: $x_{2}(n)$ processed by dual-threshold WB methods. y(n): the signals after processed by proposed method.

In our study, the artifacts in fNIRS were divided into two categories: oscillation and BS. The oscillation should be further classified into two kinds: slight oscillation and severe oscillation based on its intensity. The slight and severe oscillations in the fNIRS signals correspond to the different levels of subjects’ head movement severity, whereas the BS is generated simultaneously because of the change of head position. The artifact detection and correction approach put forward in this paper consists of artifact detection, severe oscillation correction, BS correction, slight oscillation correction, and high-pass filter, which can be described as follows (Fig. 1).

• *Motion artifact detection*. fNIRS signals were used to identify different kinds of motion artifacts including oscillations and BS.
• *Severe motion artifact processing*. Moderate and heavy oscillations, and BS removal were realized by spline interpolation.
• *Slight motion artifact removal*. Slight oscillation and the remnant motion artifacts can be dislodged with dual-threshold WB method and high-pass filtering.

#### 2.2.1 fNIRS-based detection

**Fig. 2:**
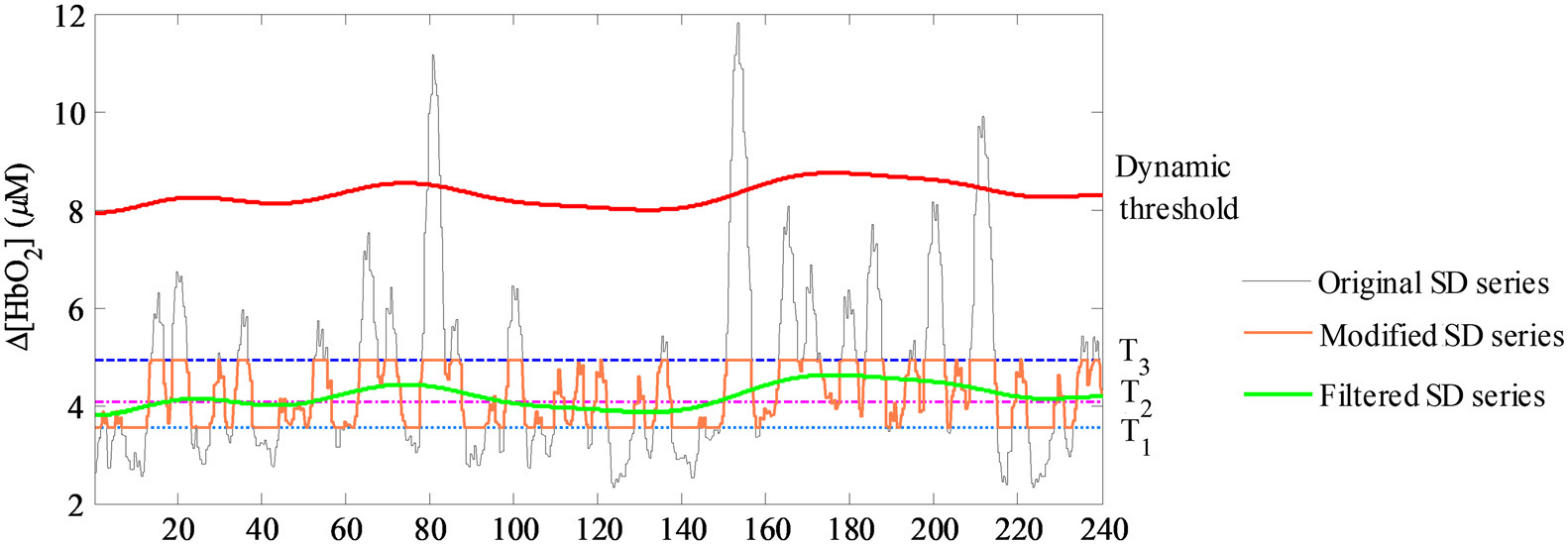
The original moving SD series t(n), the maximum value $T_{1}$, the modified moving SD series, the filtered moving SD series, and the ultimate dynamic threshold A(n).

The first step of the proposed method is the artifact detection using fNIRS-based detection strategy. fNIRS-based detection strategy uses fNIRS signal to detect oscillations and BS. Given a specific signal x(n) with sampling frequency $Fs_{\mathrm{NIRS}}$, the two-side moving SD t(n) of the measured signals can be calculated as^18^

$$t(n)=\frac{1}{W}[\overset{k}{\underset{j=-k}{\sum }}x^{2}(n+j)-\frac{1}{W}{\left(\overset{k}{\underset{j=-k}{\sum }}x(n+j)\right)}^{2}{]}^{\frac{1}{2}},$$ (3)

where n=k+1,k+2,…,N−k. N is the length of the time series x(n), and $k=3Fs_{\mathrm{NIRS}}$. W is an odd value of the sliding window’s length, and W=2k+1. A data segment for 240 s was selected from a continuous fNIRS signal that lasted 360 s to demonstrate the generation process of the ultimate dynamic threshold. By sorting the moving SD series t(n) in ascending order, p(n) is obtained. Then $T_{1}$ is defined as maximum value of the first 30% members, $T_{2}$ as maximum value of the first 50%, and $T_{3}$ as maximum value of the first 70%. Next, the data in t(n) are replaced with $T_{3}$ if exceeding $T_{3}$, and with $T_{1}$ if lower than $T_{1}$; a modified moving SD series is acquired. The modified moving SD series is filtered by a high-pass filter with a relatively low cutoff frequency of 0.02 Hz. If the data value in the filtered series is lower than $T_{2}$, it is set as $T_{2}$. Then by adding $3(T_{3}-T_{1})$, the ultimate dynamic threshold A(n) is obtained as shown in Fig. 2.

The basic criterion for oscillation detection is defined as Eq. (4). $t_{k}$ and $A_{k}$ are the corresponding data point in moving SD series t(n) and dynamic threshold series A(n), respectively. “None” means no oscillation:

$$\left\{ \begin{array}{cc} \text{if }t_{k}>2A_{k}, & \text{severe interference}\\ \text{if }A_{k}<t_{k}\leq 2A_{k}, & \text{slight interference}\\ \text{if }t_{k}\leq A_{k}, & \text{none} \end{array}.\right.$$ (4)

**Fig. 3:**
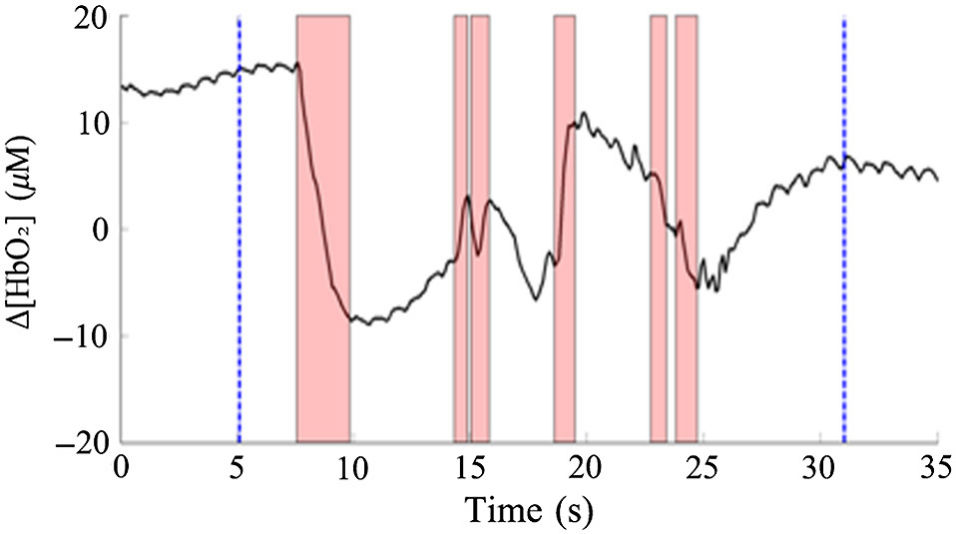
The confirmation of suspected severe oscillation. The points between the two blue dotted lines are the data with suspected severe oscillation. The shadows are confirmed as severe oscillation. The other parts between the two blue dotted lines are confirmed as slight oscillation.

As for suspected severe oscillation, further confirmation is performed. The oscillation period of fNIRS signal is divided into segments according to the slope changes. The principle is to ensure that the junction point between segments is located at the trough, and each segment contains only one peak. Another data segment for 35 s was selected to illustrate the confirmation process of the suspected severe oscillations in Fig. 3. Then the fNIRS signal range ($x_{\max}-x_{\min}$) of a certain segment is compared with the SD of the suspected oscillation area, and $x_{\max}$ and $x_{\min}$ are the maximum and minimum values of the certain segment, respectively. If $(x_{\max}-x_{\min})\geq 6\times \mathrm{SD}$, the suspected severe oscillation can be confirmed as severe oscillation, otherwise the segments of the suspected severe oscillation should be detected as slight oscillation as shown in Fig. 3.

The BS is detected using fNIRS signal based on the oscillation detection results. If the time duration of two nonoscillation segments before and after the oscillation segment in the original fNIRS signal x(n) is longer than a certain length, which is 5 s selected in our study, the mean values during the two nonoscillation segments are calculated as ${\mathrm{ave}}_{\text{before}}$ and ${\mathrm{ave}}_{\text{after}}$. $x_{\max}$ and $x_{\min}$ are the maximum and minimum values of the oscillation segment, respectively. If $|{\mathrm{ave}}_{\text{before}}-{\mathrm{ave}}_{\text{after}}|>(x_{\max}-x_{\min})/2$, a BS event could be marked for the oscillation area.

#### 2.2.2 Acceleration-based detection

In order to compare with the fNIRS-based detection, the signal from accelerometers can be used as an indicator of oscillation. First, the integrated acceleration signal acc(n) from the M channels of the accelerometer is obtained

$$\mathrm{acc}(n)=\sqrt{\frac{\sum_{k=1}^{M}a_{k}^{2}(n)}{M}}.$$ (5)

$a_{k}(n)$ is the signal series of acceleration in different directions. In this study, M=3 and $a_{k}(n)$ can represent $a_{x}(n)$, $a_{y}(n)$, and $a_{z}(n)$, respectively. The acc(n) is measured with g ($9.81{m/s}^{2}$). Next, the moving SD series d(n) of acc(n) is sorted in ascending order. And the maximum value of the first 30% of ordered d(n) is defined as T. Then the value in d(n) is compared with T. If the specific data in d(n) exceed T, then it is set as T; if lower than 0.5T, it is set as 0.5T. Then the modified moving SD series $d^{\prime }(n)$ is acquired. Finally, the dynamic threshold B(n) is obtained by doubling the $d^{\prime }(n)$. By comparing the original acceleration signals’ moving SD series d(n) with B(n), the severe and slight oscillation can be detected according to the same criterion as in fNIRS-based detection. As for BS detection, it is the same as the procedures in fNIRS-based detection.

#### 2.2.3 Severe oscillation correction using cubic spline interpolation

The cubic spline interpolation implemented in MATLAB was used to correct severe oscillation in this paper. The cubic smoothing spline function is represented as f, and the interpolation process is calculated as^5^^,^^18^

$$p\overset{N}{\underset{n}{\sum }}w(n){\left|x(n)-f(n)\right|}^{2}+(1-p)\int {\left|D^{2}f(t)\right|}^{2}dt,$$ (6)

where x(n) is the signal needed to be processed, N is the length of x(n), and $D^{2}f$ denotes the second derivative of the function f. The default value for the weight vector w(n) is all-one series. p is the smoothing parameter in the range of [0,1]. In our study, p was set to 0.99 as suggested in the previous studies.^5^^,^^18^ The cubic spline interpolation was applied to the severe oscillation area to obtain $s_{I}(n)$. After subtracting $s_{I}(n)$ from x(n), $x_{1}(n)$ was acquired.

#### 2.2.4 Baseline shift removal using spline interpolation

As for BS removal, spline interpolation method was used with a different $p=1/(Fs_{\mathrm{NIRS}}{)}^{3}$. $Fs_{\mathrm{NIRS}}$ is the sampling rate of the fNIRS signal. $s_{S}(n)$ is the signal after applying spline interpolation to the BS area. After subtracting $s_{s}(n)$ from $x_{1}(n)$ and adding the beginning DC offset value within the BS area, the signal $x_{2}(n)$ after severe artifact correction was obtained.

#### 2.2.5 Slight oscillation correction using dual-threshold wavelet-based method

Slight oscillation and the remnant artifacts can be dislodged with dual-threshold WB method. As isolated large wavelet coefficients in the discrete wavelet domain, slight artifacts can be easily identified and extracted by discrete wavelet decomposition.^16^ Applying discrete wavelet transform (DWT) to $x_{2}(n)$, wavelet coefficients can be obtained

$$w_{jk}=w_{jk}^{*}+n_{jk},\;j=1,2,\ldots ,M.$$ (7)

$w_{jk}^{*}$ is the wavelet coefficient of the interested signal. $n_{jk}$ is the wavelet coefficient of the artifacts. A detailed procedure can be found in Ref. 16. The probability of is subordinated to normal distribution, and $w_{jk}$ can be described by a mixture of Gaussians functions. That is to say, $w_{jk}∼N(0,\sigma^{2})$ and σ represents the SD of the mixed Gaussian distribution. All the $w_{jk}$ values are sorted in ascending order and numbered from 1 to L, and L represents the length of $w_{jk}$. The data point whose number is nearest to 0.1587L can be found and marked as σ^ to function as the estimation of SD of all the $w_{jk}$.

The artifacts usually result in outliers of wavelet coefficients through DWT. The artifact correction performance was compared using 16 commonly used mother wavelets. In this study, two key parameters $w_{1}$ and $w_{2}$ were proposed to determine where outliers are, which are called dual thresholds. $w_{1}$ is called detecting threshold to detect the artifacts existence. $w_{2}$ is named as processing threshold to determine whether the wavelets coefficients should be abandoned. The probability parameter α was used as a tuning parameter to determine the values of $w_{1}$ and $w_{2}$. From Eq. (8), the value of u can be calculated and used to estimate the values of $w_{1}$ and

α=2[1−Φ(−u)]. (8)

**Fig. 4:**
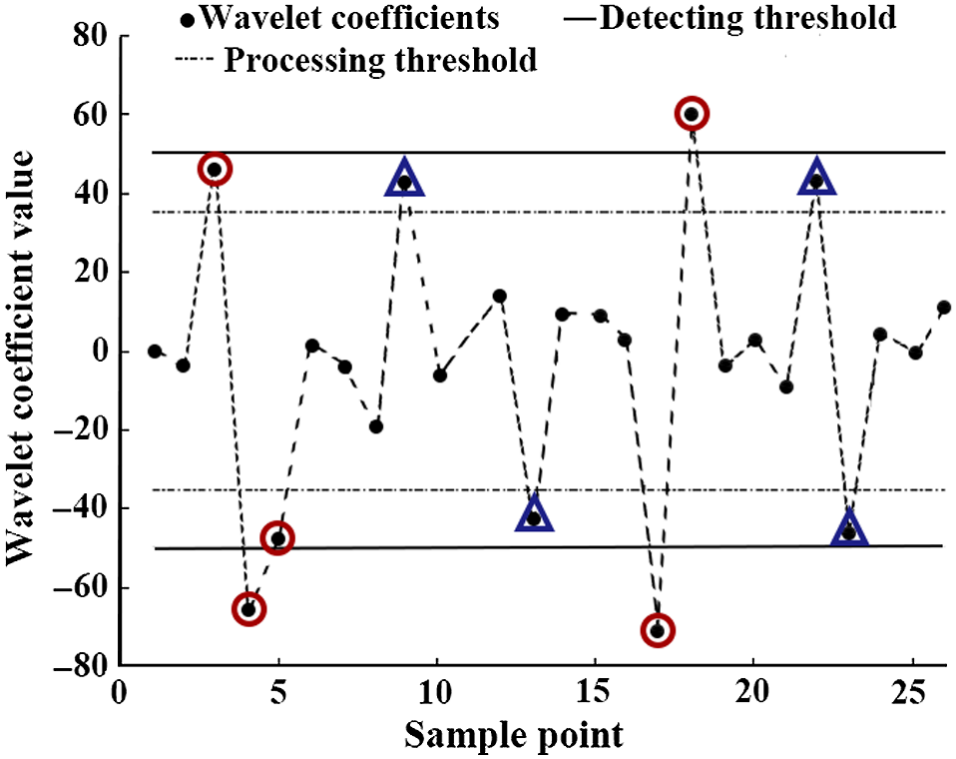
The processing of wavelet coefficients with dual-threshold WB method. The red circle areas indicate the outliers of wavelet coefficients exceeding the processing threshold should be processed. The blue triangles represent the coefficients between the two thresholds but it should not be changed because it has no neighbor whose absolute value is larger than detecting threshold.

Φ is the normal cumulative distribution function. In this study, α was set from 0.005 to 0.1 in order to explore the influence of α on the effectiveness of the proposed method. w2=u*|σ^| and $w_{1}=u*1.3|\hat{\sigma }|$. Then the absolute value of $w_{jk}$($\left|w_{jk}\right|$) is compared with $w_{1}$ according to its original order. If $\left|w_{jk}\right|$ is larger than $w_{1}$, all the near wavelet coefficients whose absolute values are larger than $w_{2}$ are found from both sides and regarded as outliers. It is stopped when meeting the first wavelet coefficient whose absolute value is lower than $w_{2}$. Then the values of all the outliers of wavelet coefficients are set to zero, and the target signal is obtained by wavelet reconstruction using processed wavelet coefficients (Fig. 4).

During the process of wavelet reconstruction, a cyclic averaging method is applied to avoid pseudo-Gibbs phenomena near singularities or abrupt changes and reduce the spike oscillation caused by the DWT as follows:

$$y_{3}(n)=\frac{1}{K}\sum_{h=1}^{K}S_{-h}(T(S_{h}(y_{2}(n)))).$$ (9)

$y_{2}(n)$ is the fNIRS signal after removal of severe oscillation, S is a shift operation, T represents the decomposition and reconstruction of discrete wavelets, and K is the number of cycles. In this paper, we selected K=16. The signals were filtered by a high-pass filter with the cut-off frequency of 0.003 Hz to remove the low-frequency oscillations. All the above algorithms used in our study were implemented in MATLAB and freely available upon request.

### 2.3 Method Comparison

To validate the effects of the proposed approach, we compared the artifact removal performance of WB, ABAMAR, spline interpolation, median filtering, Spline Savitzky–Golay (Spline SG), spline-Rloess, and severe oscillation processed with cubic spline interpolation combined with wavelet filtering, and the proposed approach including artifact detection and correction. The ABAMAR, WB, spline interpolation, and cubic spline interpolation approaches were described as above. A one-dimensional median filter (window width = 600, step = 10) was applied to the raw temporal data to eliminate outliers such as those with sudden jumps or drops and remove impulsive noise and motion artifacts. The Spline-SG method is a multistep correction procedure.^5^ A Sobel filter was first used to extract the gradient of the signal, from which time periods including motion artifacts are identified as those exceeding 1.5 times the interquartile interval. Then the BSs and slow spikes were identified and corrected those time periods using the spline approach. Finally, a Savitzky–Golay smoothing filter was performed to remove fast spikes.^5^ As for the spline-Rloess technique, the identified BSs and slow motion artifacts were also corrected by the spline approach, and the spikes were removed using Rloess filters for the spline-Rloess method. The details can be found in Ref. 27.

### 2.4 fNIRS Dataset

**Fig. 5:**
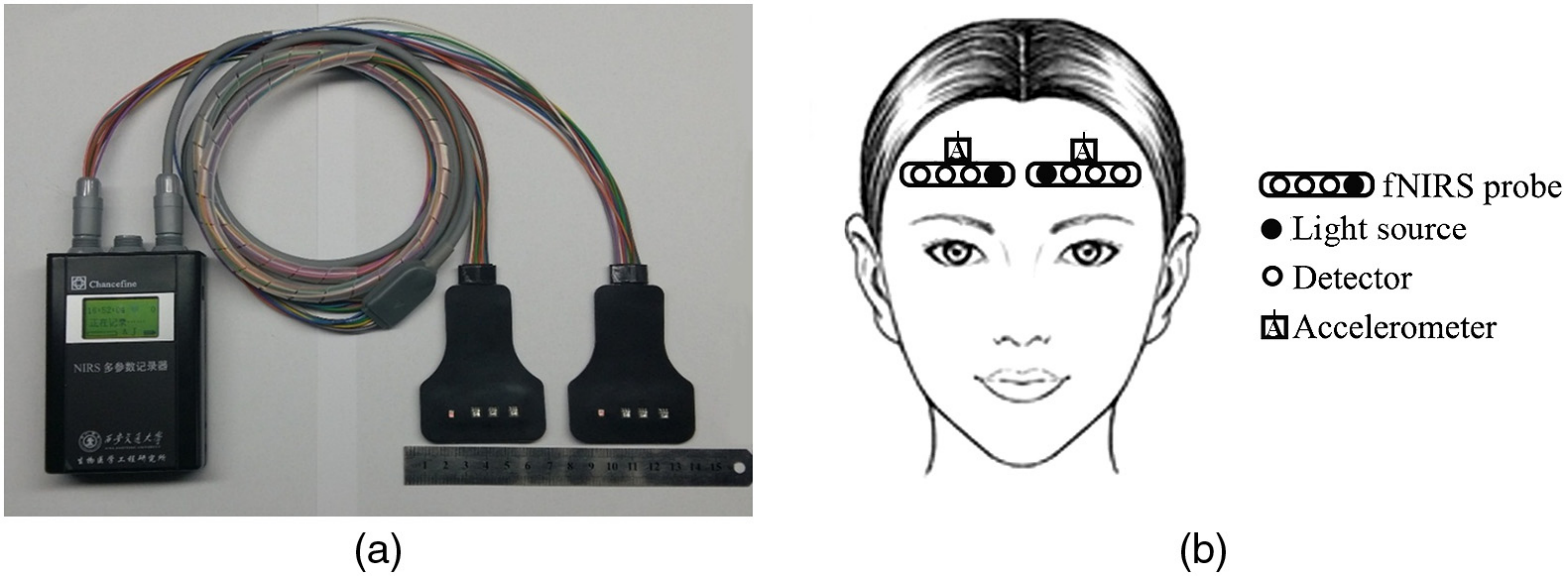
(a) The fNIRS measurement equipment including two probes. (b) The location of the near-infrared probes and accelerometers on the left and right forehead area of the subject.

In this study, a dataset was generated to evaluate the performance of the proposed approach. The study was conducted in accordance with the guidelines of declaration of Helsinki. All participants gave written informed consent. The fNIRS data were collected in the Sleep Disorders Diagnosis Center of Xijing Hospital, Military Medical University of The Air Force using a fNIRS measurement equipment developed by our laboratory to monitor the hemodynamic changes of the brain tissues at different depths in real time as shown in Fig. 5(a).^28^ The details related to the equipment can be found in Ref. 28. The near-infrared probes of the equipment were symmetrically attached on the left and right forehead area of the subjects as shown in Fig. 5(b). The near-infrared signals were converted into the blood oxygen signals including surface deoxygenated hemoglobin (deoxy-Hb), surface oxygenated hemoglobin (oxy-Hb), proximal deoxy-Hb, proximal oxy-Hb, distal deoxy-Hb, distal oxy-Hb with units of μmol/L, and local tissue blood oxygen saturation with a unit of percentage. An accelerometer was attached to each fNIRS probe to record the acceleration. The sampling frequency was 10 Hz. Forty healthy participants (16 male and 24 females, mean age 32 years) were involved in this study. The subjects were asked to sleep the whole night (approximately from 11:00 pm to 6:30 am) as usual until they were awoken by the researcher monitoring the measurements. Involuntary movements of the subjects during the whole night were not restricted.

According to the accelerometer’s signals, a segment (totally lasted 1.5 h) of relatively clean distal signals (${\Delta [\mathrm{HbO}}_{2}]$) was selected as a criterion for the performance evaluation. Then 60 different segments (each lasted 1.5 h) of noise polluted ${\Delta [\mathrm{HbO}}_{2}]$ signals were chosen from all the subjects by visual interpretation according to the accelerometer’s signal recordings, in order to extract the noise signals. The accelerometer-based method^8^ combined with WB method^16^ and a 0.002-Hz high-frequency filter were applied to remove the high-frequency spike-like artifacts, low-frequency artifacts, and also the BSs in the signal. The processed signals were subtracted from the original signals to acquire the noise signals. Finally, 60 simulated segments of ${\Delta [\mathrm{HbO}}_{2}]$ signals, each lasted 1.5 h, were generated by combining the selected clean signal with the 60 noise segments. A sample simulated time course without the artifact, with uncorrected artifacts, and corresponding corrected sample time course without the artifacts detection strategy, with the acceleration-based detection strategy, and with the fNIRS-based detection strategy were compared to validate the effects of artifact detection strategy. Finally, the comparison methods including WB, ABAMAR, spline interpolation, median filtering, Spline SG, spline-Rloess, and severe oscillation processed with cubic spline interpolation combined with wavelet filtering, and the proposed approach were applied to all of these data.

### 2.5 Evaluation Metrics

Two metrics, SNR and Pearson’s correlation coefficient (R), were calculated to evaluate the effect of the artifact detection and correction method. The SNRs for the simulated noised signal and the processed signal after artifact correction are defined as ${\mathrm{SNR}}_{\mathrm{noised}}$ and ${\mathrm{SNR}}_{\text{processed}}$, respectively. Assuming that the clean signal is x(n),n=1,…,N, and X(n) is the noised signal, N(n)=X(n)−x(n), where N(n) is the noise signal. The ${\mathrm{SNR}}_{\mathrm{noised}}$ for the simulated noised signal X(n) is defined as

$${\mathrm{SNR}}_{\text{noised}}=10\;\log_{10}\frac{\sum {\left|x(n)\right|}^{2}}{\sum {\left|X(n)-x(n)\right|}^{2}}.$$ (10)

As for the processed signals, y(n) is the processed signal after the artifact removal, and N(n)=y(n)−x(n). The ${\mathrm{SNR}}_{\text{processed}}$ for the processed signal y(n) is defined as

$${\mathrm{SNR}}_{\text{processed}}=10\;\log_{10}\frac{\sum {\left|x(n)\right|}^{2}}{\sum {\left|y(n)-x(n)\right|}^{2}}.$$ (11)

The R represents the similarity between the clean signal x(n) and the processed signal y(n), which is calculated as follows:

$$R=\frac{\overset{N}{\underset{n=1}{\sum }}[x(n)-\overset{¯}{x}][y(n)-\overset{¯}{y}]}{\sqrt{\overset{N}{\underset{n=1}{\sum }}{\left[x(n)-\overset{¯}{x}\right]}^{2}\cdot \overset{N}{\underset{n=1}{\sum }}{\left[y(n)-\overset{¯}{y}\right]}^{2}}}.$$ (12)

## 3 Results

### 3.1 Effects of Probability Parameter and Mother Wavelet for Dual-Threshold Wavelet-Based Method

**Fig. 6:**
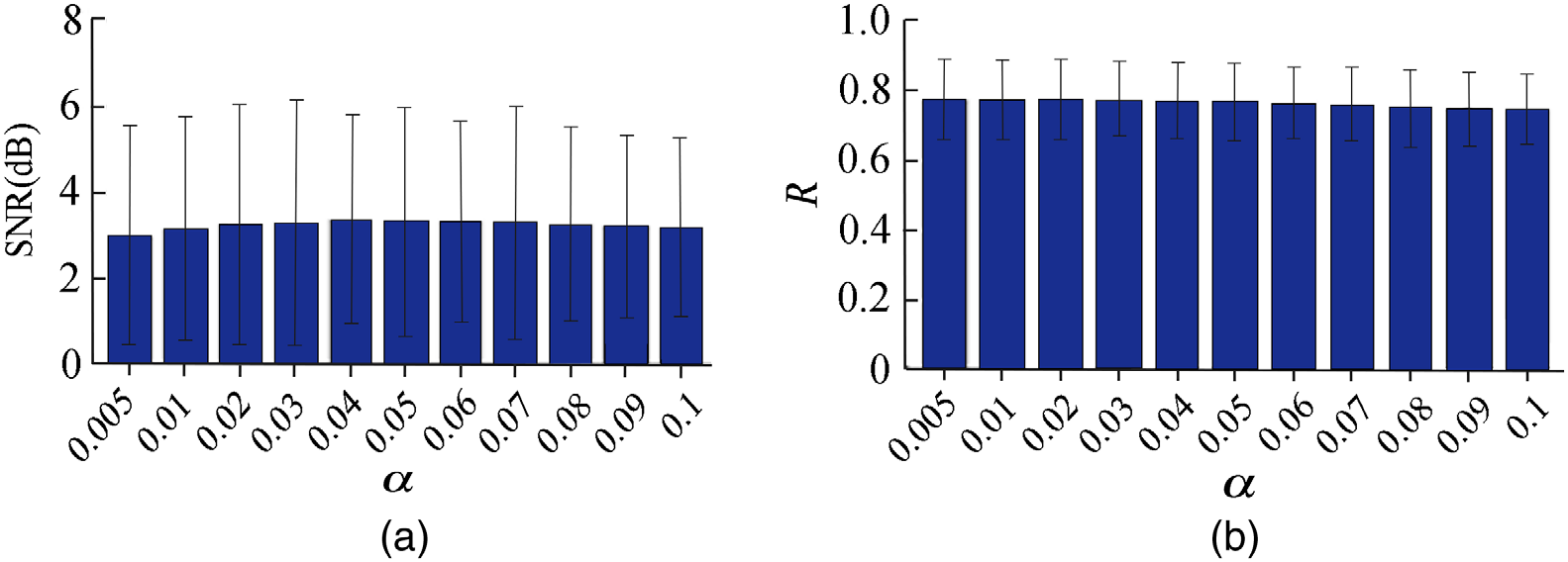
The (a) SNR and (b) R results while α changes from 0.005 to 0.1.

The effect of probability parameter α ranging from 0.005 to 0.1 was assessed for dual-threshold WB method on the performance of the proposed method as shown in Fig. 6. It indicates that the SNR and R value is almost the same, whereas α changes from 0.005 to 0.1. There is no significant difference on the SNR and R for α value from 0.005 to 0.1.

**Fig. 7:**
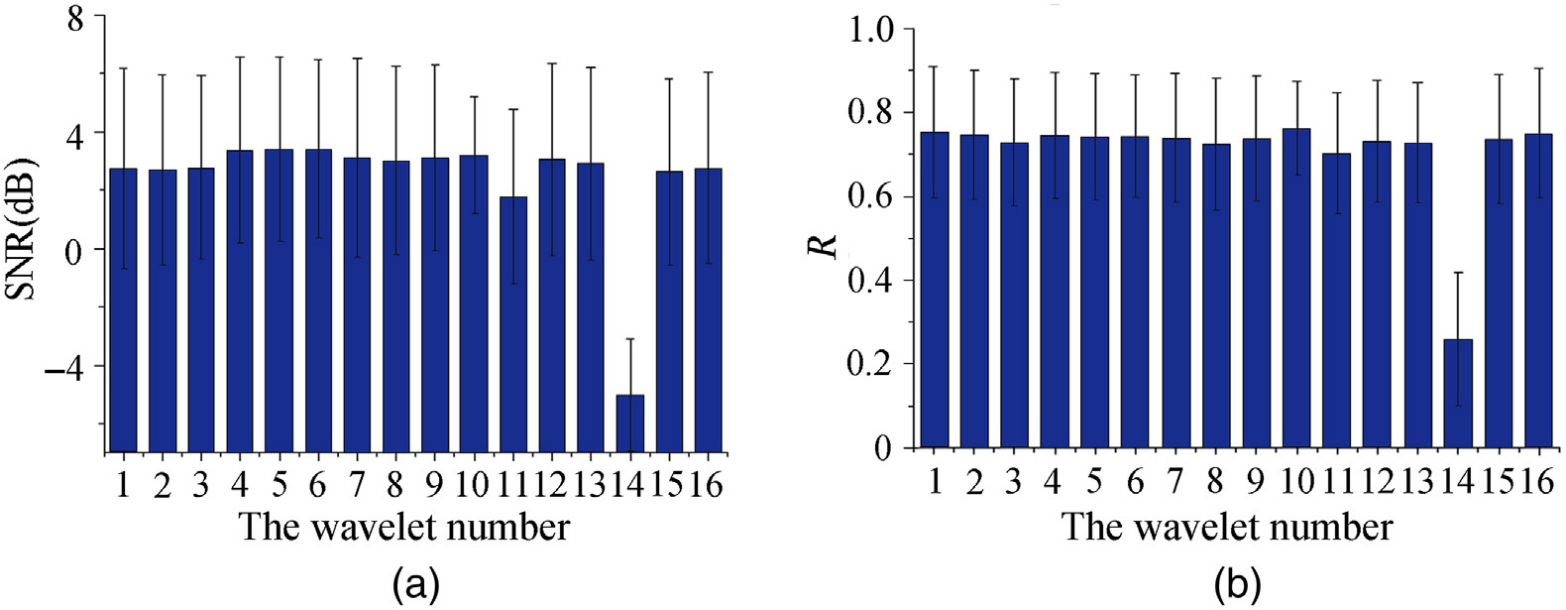
The performance comparison results of 16 types of different mother wavelets by (a) SNR and (b) R.

The artifact correction performance was compared using 16 commonly used mother wavelets for dual-threshold WB method. The mother wavelet types from number 1 to 16 are shown in Table 1. Figure 7 demonstrates that only one type of mother wavelet (rbio3.3) performed relatively poor. There are significant differences on the SNR and R between rbio3.3 and other mother wavelets, whereas there is no significant difference for the other 15 types of mother wavelets.

**Table 1:** The mother wavelet types.

No.	Type	No.	Type	No.	Type	No.	Type
1	db3	5	coif4	9	sym5	13	bior5.5
2	db4	6	coif5	10	dmey	14	rbio3.3
3	db5	7	sym3	11	bior3.3	15	rbio4.4
4	coif3	8	sym4	12	bior4.4	16	rbio5.5

**Fig. 8:**
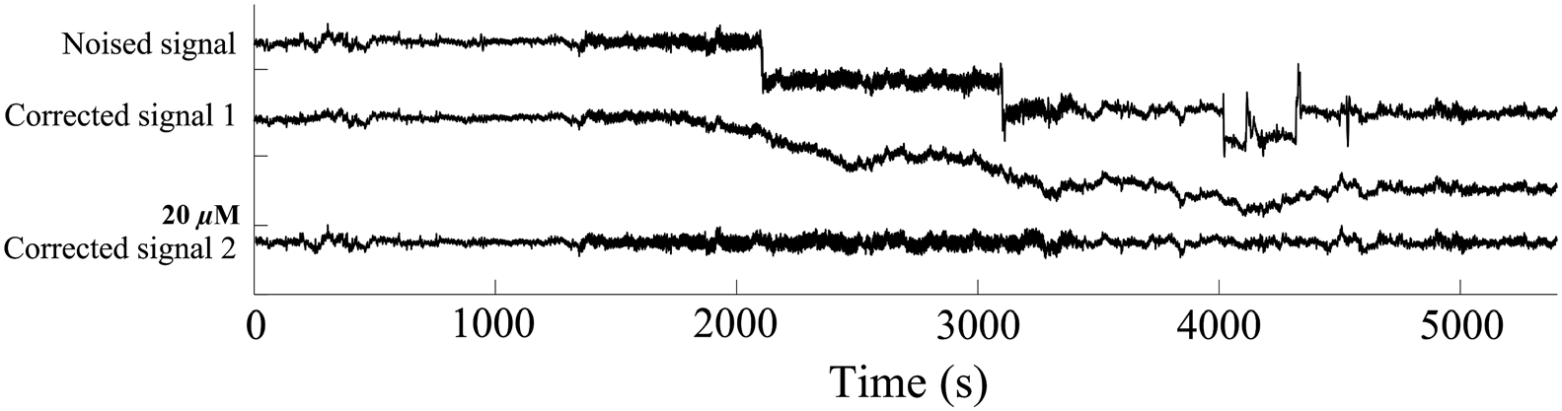
Comparison of motion artifact removal results between wavelet filtering and dual threshold WB method. Corrected signal 1: artifacts removed signal with wavelet filtering. Corrected signal 2: artifacts removed signal with dual-threshold WB method.

Figure 8 shows typical results of applying the dual-threshold WB method and wavelet filtering to a synthesized signal, respectively. The synthesized signal contains high-frequency spike-like artifacts, BSs, and the amplitude changes with time. The dual-threshold WB method has the capability to remove those artifacts, and the wavelet filtering is poor for BSs.

### 3.2 Artifact Detection Strategies Comparison

**Fig. 9:**
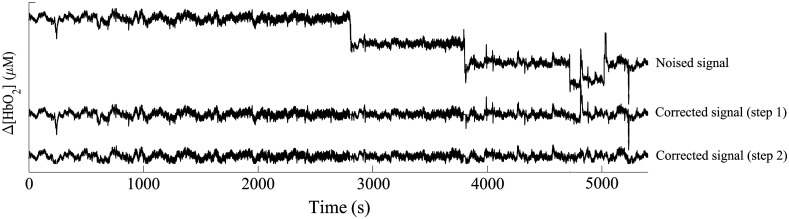
A typical simulated wave and the corresponding waves obtained at each step. Corrected signal (step 1): severe oscillation and BS removal signal. Corrected signal (step 2): slight oscillation removal signal.

**Fig. 10:**
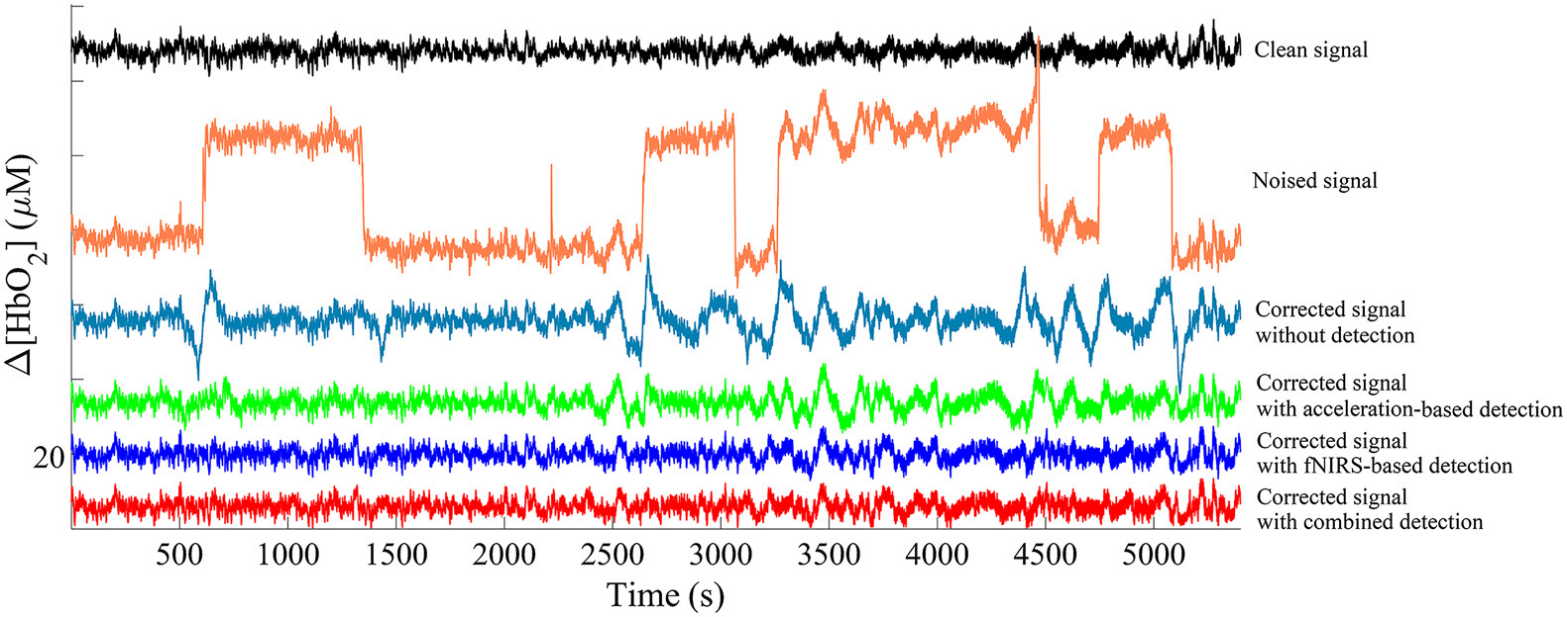
The time courses of a typical example of the clean signal, the artifact polluted signal, and corresponding corrected signal without the artifact detection strategy, with the acceleration-based detection strategy, and with the fNIRS-based detection strategy, respectively.

A typical simulated wave and the corresponding waves obtained after severe oscillation and BS correction, and slight oscillation correction is shown in Fig. 9. A sample simulated time course without the artifacts, with uncorrected artifacts, and corresponding corrected sample time course without the artifact detection strategy, with the acceleration-based detection strategy, with the fNIRS-based detection strategy, and with the combination of acceleration- and fNIRS-based detection strategy are shown in Fig. 10, respectively. For the corrected sample time course without the artifact detection strategy, severe motion artifacts were not processed and only WB method was applied. Compared with the noised signal, the quality of the corrected signal was significantly improved through the artifact correction. Taking the clean signal as a reference, the corrected signal with the fNIRS-based detection strategy and with the combined strategy behaved best, nearly as good as clean data in contrast with the corrected signal without the artifact detection strategy and with the acceleration-based detection strategy. There is no significant difference between the corrected signals with the fNIRS-based strategy and the combined strategy (P>0.05). Thus the fNIRS-based detection strategy is selected in our study.

**Fig. 11:**
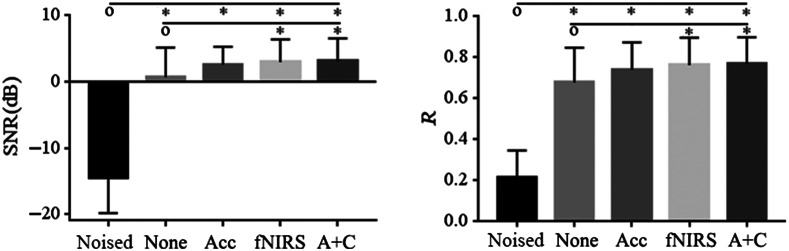
The artifact correction performance comparison evaluated by SNR and R between the uncorrected signal, corrected signal without the artifact detection strategy, corrected signal with the acceleration-based detection strategy, and the corrected signal with fNIRS-based detection strategy. “o” represents the basic method; * means P<0.05/7.

The simulated noised signal was compared with the corrected signal without detection strategy, with the acceleration-based detection strategy, with the fNIRS-based detection strategy, and also with the combined detection strategy, respectively. And the corrected signal without detection strategy was also compared with the signal with the acceleration-based detection strategy, with the fNIRS-based detection strategy, and with combined detection strategy, respectively. The statistical analysis results across all the 60 fNIRS segments by separate paired t-test with Bonferroni correction are shown in Fig. 11. The mean and SD of the SNR and R for the corrected signal with the fNIRS-based detection strategy and with the combined detection strategy are almost the same. There are significant differences on the SNR and R between the corrected signal without detection strategy and with fNIRS-based detection strategy and also with the combined detection strategy (P<0.05/7).

### 3.3 Artifact Correction Methods Comparison Results

**Fig. 12:**
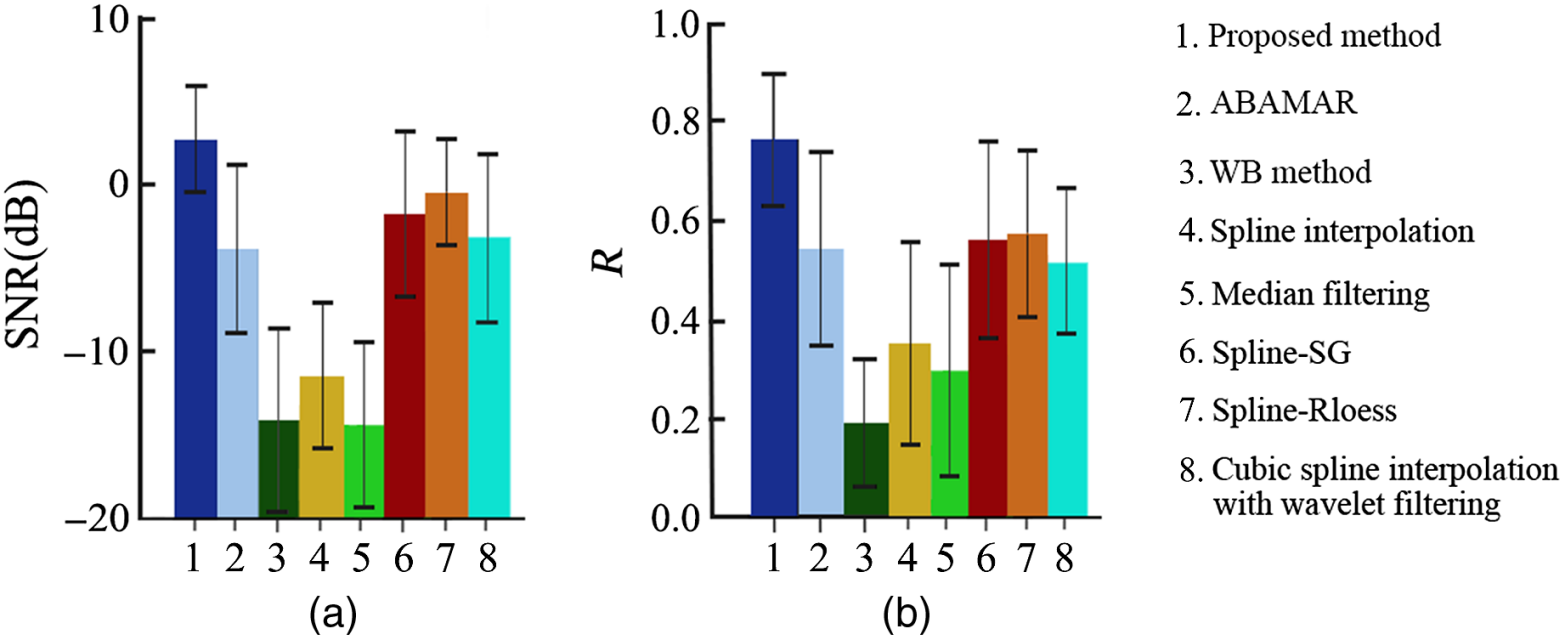
The performance comparison of the proposed method with different approaches, including WB, ABAMAR, spline interpolation, median filtering, Spline SG, spline-Rloess, and severe oscillation processed with cubic spline interpolation combined with wavelet filtering, by (a) SNR and (b) R.

The artifact correction performance of the proposed method compared with different approaches, including WB, ABAMAR, spline interpolation, median filtering, Spline SG, spline-Rloess, and severe oscillation processed with cubic spline interpolation combined with wavelet filtering, are shown in Fig. 12. We could see the proposed method performed best both in the SNR and R. Statistical analysis was performed between the proposed method and other approaches including WB, ABAMAR, spline interpolation, median filtering, Spline SG, spline-Rloess, and severe oscillation processed with cubic spline interpolation combined with wavelet filtering, respectively. Separate paired t-test with Bonferroni correction for multiple comparison was applied to compare the proposed method with other approaches. The results proved there are significant differences on the SNR (WB, spline interpolation, and median filtering: P<0.05/7) and R (WB, spline interpolation, and median filtering: P<0.05/7) between the proposed method and other approaches.

## 4 Discussion

In this study, an optimum algorithm for the artifact detection and correction was explored for fNIRS data. We proposed a hybrid artifact detection and correction approach here. First, we applied a distinct artifact detection method through fNIRS artifact detection strategy solely based on the variation in the fNIRS signal. Next, we developed a comprehensive correction through three main steps: severe artifact correction by cubic spline interpolation, BS removal by spline interpolation, and slight oscillation reduction by dual-threshold WB method. In order to assess the performance of the proposed novel hybrid approach, we have compared the proposed method with other commonly used approaches, and the results show that this approach yields better performance in artifact detection and correction than other methods and nearly as good as clean data.

The artifacts directly influence the optical signals and ultimately hemodynamic signals through calculating process. We hypothesized that the artifact removal with the proposed approach could be applied to hemodynamic signals directly, and it can also be used in optical signals and then obtains the hemodynamic signals due to the fact that the motion artifacts, low-frequency drift, and BSs should be visible at the OD level. The theoretical calculation process of hemodynamic signals from optical signals is shown in Eqs. (1) and (2), and the $\alpha_{1}$, $\alpha_{2}$, $\beta_{1}$, and $\beta_{2}$ are calculated as

$$\alpha_{1}=\frac{\varepsilon_{{\mathrm{HbO}}_{2}}^{\lambda_{2}}}{D_{\mathrm{PF}}^{\lambda_{1}}(\varepsilon_{\mathrm{Hb}}^{\lambda_{1}}\varepsilon_{{\mathrm{HbO}}_{2}}^{\lambda_{2}}-\varepsilon_{\mathrm{Hb}}^{\lambda_{2}}\varepsilon_{{\mathrm{HbO}}_{2}}^{\lambda_{1}})},\;\alpha_{2}=\frac{\varepsilon_{{\mathrm{HbO}}_{2}}^{\lambda_{1}}}{D_{\mathrm{PF}}^{\lambda_{2}}(\varepsilon_{\mathrm{Hb}}^{\lambda_{1}}\varepsilon_{{\mathrm{HbO}}_{2}}^{\lambda_{2}}-\varepsilon_{\mathrm{Hb}}^{\lambda_{2}}\varepsilon_{{\mathrm{HbO}}_{2}}^{\lambda_{1}})},$$ (13)

$$\beta_{1}=\frac{\varepsilon_{{\mathrm{HbO}}_{2}}^{\lambda_{2}}}{D_{\mathrm{PF}}^{\lambda_{1}}(\varepsilon_{\mathrm{Hb}}^{\lambda_{1}}\varepsilon_{{\mathrm{HbO}}_{2}}^{\lambda_{2}}-\varepsilon_{\mathrm{Hb}}^{\lambda_{2}}\varepsilon_{{\mathrm{HbO}}_{2}}^{\lambda_{1}})},\;\beta_{2}=\frac{\varepsilon_{\mathrm{Hb}}^{\lambda_{1}}}{D_{\mathrm{PF}}^{\lambda_{2}}(\varepsilon_{\mathrm{Hb}}^{\lambda_{1}}\varepsilon_{{\mathrm{HbO}}_{2}}^{\lambda_{2}}-\varepsilon_{\mathrm{Hb}}^{\lambda_{2}}\varepsilon_{{\mathrm{HbO}}_{2}}^{\lambda_{1}})}.$$ (14)

$\varepsilon_{{\mathrm{HbO}}_{2}}^{\lambda_{1}}$ and $\varepsilon_{{\mathrm{HbO}}_{2}}^{\lambda_{2}}$ are the extinction coefficients of oxygenated hemoglobin at the two wavelengths; $\varepsilon_{\mathrm{Hb}}^{\lambda_{1}}$ and $\varepsilon_{\mathrm{Hb}}^{\lambda_{2}}$ are the extinction coefficients of deoxygenated hemoglobin; $D_{\mathrm{PF}}^{\lambda_{1}}$ and $D_{\mathrm{PF}}^{\lambda_{2}}$ are the differential path factors of the two wavelength, which represents the average length of the path photons travels in the human tissue; $\Delta {\mathrm{OD}}^{\lambda_{1}}$ and $\Delta {\mathrm{OD}}^{\lambda_{2}}$ are the optical density of the two wavelengths, which can be described as

$$\mathrm{OD}=-\log\;\frac{I}{I_{0}}=\varepsilon {\mathrm{CLD}}_{\mathrm{PF}}+G,$$ (15)

where $I_{0}$ is the input light intensity, I is the detected light intensity, ε is the extinction coefficients of the tissue, C is the concentration, and G is the absorption factor of the background. The coefficients $\alpha_{1}$, $\alpha_{2}$, $\beta_{1}$, and $\beta_{2}$ vary with the used wavelength of near infrared light. The wavelengths of our fNIRS measurement are 735 and 850 nm. The corresponding values of the coefficients are $\alpha_{1}=330.1717$, $\alpha_{2}=-131.3958$, $\beta_{1}=-127.6967$, and $\beta_{2}=184.5598$, respectively. Generally, $\Delta {\mathrm{OD}}^{\lambda_{1}}$ and $\Delta {\mathrm{OD}}^{\lambda_{2}}$ are synchronized and they usually have the same sign. And the hemodynamic signals calculated from the optical measurement will result in about $200a_{1}$ (for $\Delta [{\mathrm{HbO}}_{2}]$) and $60a_{2}$ (for Δ[Hb]) with the unit of μmol/L($a_{1}$ and $a_{2}$ represent certain constants). However, when applying the proposed approach to optical measurement to remove the motion artifacts, the procedures of the different optical signals in different wavelengths are relatively independent, so the processed optical signals in different wavelengths may be asynchronous among the oscillation areas. If the $\Delta {\mathrm{OD}}^{\lambda_{1}}$ and $\Delta {\mathrm{OD}}^{\lambda_{2}}$ are of the opposite sign, the hemodynamic signals calculated from the optical measurement will result in about $460a_{1}$ (for $\Delta [{\mathrm{HbO}}_{2}]$) and $300a_{2}$ (for Δ[Hb]) with the unit of μmol/L, and they all diverge from the regular results. It can be observed that Δ[Hb] will be more than 5 times larger than that of the same sign, but only about 2 times of $\Delta [{\mathrm{HbO}}_{2}]$. Then the hemodynamic signals calculated from the optical measurements might diverge from the regular results within the artifacts segments because of the effects of the coefficients. Although this divergence might rarely happen, we recommend applying the artifact removal approach to hemodynamic signals directly rather than to optical signals.

In single-threshold WB method, the value of the threshold will directly influence the number of outliers, resulting in the differences in the artifact correction effect.^15^^,^^16^ However, a constant threshold value might not satisfy the requirements of all the signals. In this study, we analyzed the influence of the threshold in dual-threshold WB methods by comparing the results for different values of the threshold from 0.005 to 0.1. It can be found in Fig. 6 that the influence of the different on the performance of the proposed method is not significant both in SNR and R. In fact, the effect of varying from 0.005 to 0.2 on SNR and R was tested (the result is not shown in this paper). The results also show that proposed method is not sensitive to the change of. So the threshold changes will not directly influence the artifact correction effects for the dual-threshold WB method proposed in our study. The reason might be the interval between the detecting threshold and processing threshold makes the result of the artifactWe compared artifacts removal correction is not so sensitive to the threshold changes for the signals with different qualities. Thus the disadvantages of the single-threshold WB method can be overcome in the dual-threshold approach. It can be inferred that the dual-threshold approach can locate the artifacts exactly for the fNIRS signal with motion artifacts and avoid useful information be abandoned to some extent for the relatively clean signals.

Mother wavelet also performs an important role in DWT. We compared artifact removal performance of 16 different mother wavelets in our study as shown in Fig. 7. In order to keep the valid frequency band of interest (0.003 to 0.04 Hz)^29^^,^^30^ and discard the low-frequency oscillation, which is the offset after BS removal, a high-pass filter with cutoff frequency at 0.003 Hz was used. We found that among 16 commonly used mother wavelets only one (rbio3.3) performed relatively poor. It revealed that the wavelet denoising effect of the proposed method in our study is not sensitive to the type of the mother wavelets.

We analyzed the performance of artifact removal with different artifact detection strategies. Taking the clean signal as a reference, it can be concluded that the corrected signal with the fNIRS-based detection strategy and with the combined strategy performed best, almost as good as clean data compared with the corrected signal without the artifact detection strategy and with the acceleration-based detection strategy as shown in Fig. 11. There is no significant difference between the corrected signals with the fNIRS-based strategy and the combined strategy. The acceleration-based detection strategy performed relatively poorer because not every fluctuation in acceleration signals corresponded to an oscillation or BS in the same position of fNIRS signals. So the acceleration-based detection strategy may result in a loss of some useful information. Similarly, not every fluctuation in fNIRS signals was caused by the motion. To make it simpler, we recommend detecting the artifacts based on fNIRS signals. Thus it could work even if there are no acceleration signals.

As we can see from Fig. 12, the proposed method performed better than WB, ABAMAR, spline interpolation, median filtering, spline, spline-SG, spline-Rloess, and severe oscillation processed with cubic spline interpolation combined with wavelet filtering method both in the SNR and R. Although Brigadoi et al.^20^ reported that WB method was the most effective method to remove the low-frequency and low-amplitude artifacts compared with other methods, some researchers found that it might be inability to correct lasting shift when applied to fNIRS data, which contain various artifacts especially BSs.^15^ We can see from Fig. 10 that WB method performance was worst among all the techniques. It might be caused by the fact that WB method cannot deal with large BSs. The application of dynamic threshold and segmentation in the artifact detection in our proposed method can locate the different types of the artifacts more accurately, then the artifact correction algorithm can be adopted to match the artifacts with different types. Furthermore, WB method using the median value of wavelet coefficients to estimate the SD is not reasonable especially when the signal’s quality was extremely poor. WB method imposed a single Gaussian distribution on wavelet coefficients. As for the dual-threshold WB method in the proposed method, we improved the way to estimate the SD of the Gaussian distribution and introduced the concept of dual threshold. The distribution of wavelet coefficients can be described by a mixture of Gaussians.^31^^,^^32^ Additionally, the effectiveness of WB method is sensitive to the threshold value.^15^ In contrast, the dual threshold in the proposed method is appropriate to fNIRS signals in different qualities, and it can perform well with different thresholds as mentioned above. The ABAMAR performs well at the correction of BS, but it uses the mean value in place of the whole data segments with the artifacts, which ignores some valid information. The use of spline interpolation method can overcome the problem, which could correct the artifacts as well as reserve the useful information. The drawback of this approach occurs if the artifacts are difficult to detect.^20^ The performance of spline-SG, spline-Rloess, and severe oscillation processed with cubic spline interpolation combined with wavelet filtering method is similar to the ABAMAR both in the SNR and R in our study. The Rloess and SG methods could remove high-frequency motion artifacts with relatively weak performance in correcting slow motion artifacts or BSs.^5^

The proposed approach demonstrated effective performance on the removal of motion artifacts and BS. On the other hand, the noises and artifacts in fNIRS measurements are very challenging, and they might be from various sources with distinct statistical properties. For example, instrument noise, such as electronic (Johnson–Nyquist) noise and shot noise, may be induced by hardware and surrounding space with a Poisson distribution.^33^ Low-frequency drift may be introduced by small instabilities in the laser diode light sources. Measurement noise is assumed to be Gaussian white noise. Finally, signal variation introduced by the human subject may include motion artifact and “global” oscillations from systemic sources (pulse, respiration, blood pressure Mayer waves, and other low-frequency oscillations), as well as the localized hemodynamic response to task-induced brain activation. Since photons reflected from brain tissue must travel through the skin, skull, and dura before reaching a detector on the scalp surface, there is also oscillation in the fNIRS signal arising from fluctuations in the superficial layers of the scalp.^14^ Therefore, it is hard to say that the artifacts can be totally removed by pure signal processing.

In conclusion, considering the disadvantages of the previous approaches, the proposed approach provides a distinct detection of the artifacts through fNIRS detection strategy, and a comprehensive correction through three main steps: severe artifact correction by cubic spline interpolation, BS removal by spline interpolation, and slight oscillation reduction by dual-threshold WB method.
